# Comparative analysis of fungal genomes reveals different plant cell wall degrading capacity in fungi

**DOI:** 10.1186/1471-2164-14-274

**Published:** 2013-04-23

**Authors:** Zhongtao Zhao, Huiquan Liu, Chenfang Wang, Jin-Rong Xu

**Affiliations:** 1 NWAFU-PU Joint Research Center and State Key Laboratory of Crop Stress Biology for Arid Areas, College of Plant Protection, Northwest A&F University, Yangling, Shaanxi 712100, China; 2 Department of Botany and Plant Pathology, Purdue University, West Lafayette, IN, 47907, USA

**Keywords:** Fungi, CAZymes, Glycoside hydrolase, Polysaccharide lyase, Carbohydrate esterase, Pectinase, Cutinase, Lignocellulase

## Abstract

**Background:**

Fungi produce a variety of carbohydrate activity enzymes (CAZymes) for the degradation of plant polysaccharide materials to facilitate infection and/or gain nutrition. Identifying and comparing CAZymes from fungi with different nutritional modes or infection mechanisms may provide information for better understanding of their life styles and infection models. To date, over hundreds of fungal genomes are publicly available. However, a systematic comparative analysis of fungal CAZymes across the entire fungal kingdom has not been reported.

**Results:**

In this study, we systemically identified glycoside hydrolases (GHs), polysaccharide lyases (PLs), carbohydrate esterases (CEs), and glycosyltransferases (GTs) as well as carbohydrate-binding modules (CBMs) in the predicted proteomes of 103 representative fungi from *Ascomycota, Basidiomycota, Chytridiomycota*, and *Zygomycota*. Comparative analysis of these CAZymes that play major roles in plant polysaccharide degradation revealed that fungi exhibit tremendous diversity in the number and variety of CAZymes. Among them, some families of GHs and CEs are the most prevalent CAZymes that are distributed in all of the fungi analyzed. Importantly, cellulases of some GH families are present in fungi that are not known to have cellulose-degrading ability. In addition, our results also showed that in general, plant pathogenic fungi have the highest number of CAZymes. Biotrophic fungi tend to have fewer CAZymes than necrotrophic and hemibiotrophic fungi. Pathogens of dicots often contain more pectinases than fungi infecting monocots. Interestingly, besides yeasts, many saprophytic fungi that are highly active in degrading plant biomass contain fewer CAZymes than plant pathogenic fungi. Furthermore, analysis of the gene expression profile of the wheat scab fungus *Fusarium graminearum* revealed that most of the CAZyme genes related to cell wall degradation were up-regulated during plant infection. Phylogenetic analysis also revealed a complex history of lineage-specific expansions and attritions for the PL1 family.

**Conclusions:**

Our study provides insights into the variety and expansion of fungal CAZyme classes and revealed the relationship of CAZyme size and diversity with their nutritional strategy and host specificity.

## Background

Carbohydrate-active enzymes (CAZymes) are responsible for the breakdown, biosynthesis or modification of glycoconjugates, oligo- and polysaccharides. Most importantly, the CAZymes produced by parasites play a central role in the synthesis and breakdown of plant cell wall as well as in host-pathogen interactions [1]. At present, the CAZymes have been grouped into four functional classes: glycoside hydrolases (GHs), glycosyltransferases (GTs), polysaccharide lyases (PLs), and carbohydrate esterases (CEs) based on their structurally-related catalytic modules or functional domains [1]. Among them, the CAZymes of classes CE, GH, and PL are often known as cell wall degrading enzymes (CWDEs) due to their important roles in plant biomass decomposition by fungi and bacteria [2]. In addition to the catalytic modules, around 7% of CAZymes also contain the carbohydrate-binding modules (CBMs), which are the most common non-catalytic modules associated with enzymes active in cell-wall hydrolysis [1].

Fungi can produce all kinds of CAZymes [1,3]. Among them, plant cell wall degrading enzymes received special attentions because of their importance in fungal pathogens for penetration and successful infection of their hosts. Carbohydrates released from plant cell wall also can supply nutrition for fungal growth. As a matter of fact, some saprophytic fungi obtain nutrition for growth and reproduction mainly by degrading plant cell wall materials with a variety of CWDEs. A number of studies have revealed that activities of hydrolytic enzymes from different fungi showed preferences for different types of plant biomass and adaption to their lifestyles [4,5]. When cultured on different substrates, various plant biomass degrading enzymes were shown to be produced by different fungi, including the model filamentous fungus *Neurospora crassa*[6-12]. The white-rot basidiomycete fungi such as *Phanerochaete chrysosporium* are found to be the main producers of ligninases for substantial lignin decay in wood [13,14]. For fungal pathogens, localized degradation of cell wall is necessary for accessing plant cytoplasm and spreading across host tissues. In several plant pathogenic fungi, CWDEs such as pectinases and xylanases were demonstrated to be related to pathogenicity or virulence [15-17].

To date, over a hundred of fungal genomes have been sequenced and are publicly available, including representative fungi from *Ascomycota, Basidiomycota, Zygomycota,* and *Chytridiomycota*. Most of fungi except Saccharomycetes and Schizosaccharomycetes have a large number of CWDE genes that are likely involved in plant infection or survival in the environments. Some genes coding polysaccharide degrading enzymes have expanded family members in certain fungi and gene redundancy has been shown to guard critical functions [18]. However, a complete and systematic comparative analysis of CAZymes across the fungal kingdom has not been reported. In addition, it is still unclear whether the distribution of CAZymes in fungi is related to the plant cell wall components, although plant cell walls of dicots and monocot are known to be composed of different components particularly on pectins and hemicelluloses [5,19,20].

In this study, we identified and compared the full repertoires of CAZymes from representative fungi and performed a comprehensive comparison upon the distribution and abundance of CAZyme families to obtain clues to their digestive potential, especially against plant cell wall polysaccharides. Differences in the number and variety of CAZymes among saprophytic, facultative parasitic, hemi-biotrophic, biotrophic, and symbiotic fungi were analyzed. The relationship between the number and variety of CAZymes and fungal nutritional strategy and host specificity was also examined.

## Results and discussion

### The distribution of CAZyme families

The predicted proteomes of 103 fungi from *Ascomycota*, *Basidiomycota*, *Chytridiomycota*, and *Zygomycota* were systematically screened for different families of CAZymes and CBMs based on family-specific HMMs [21]. These fungi represent five types of nutritional mode, saprophytic, facultative parasitic, hemi-biotrophic, biotrophic, and symbiotic fungi, and include pathogens of plants, vertebrates, nematodes, and insects.

**Figure 1:**
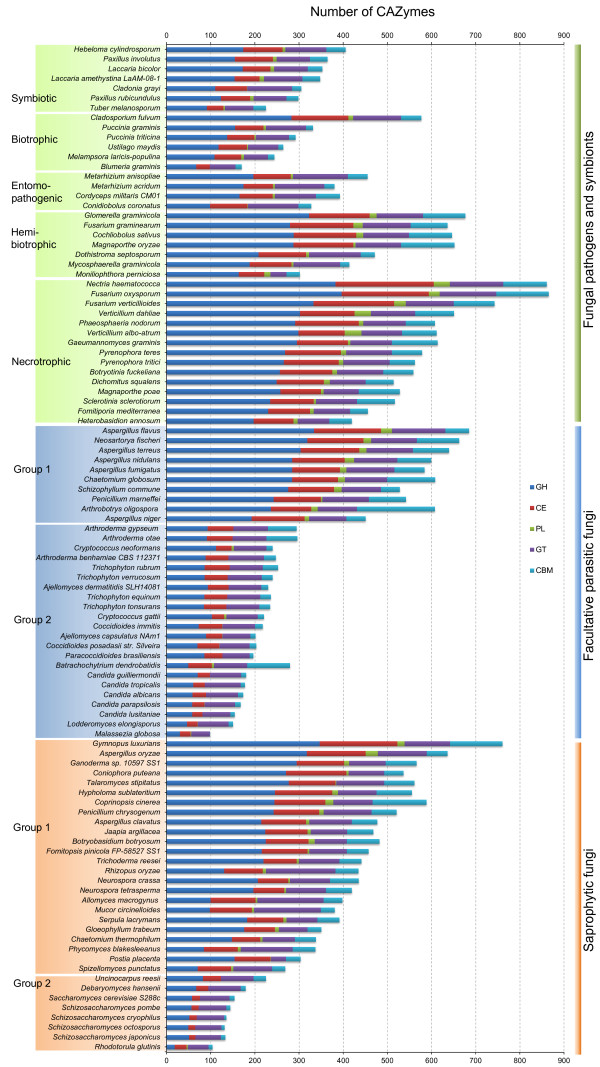
**Comparative analysis of fungal CAZymes. **The numbers of CAZyme modules or domains were represented as horizontal bars. CBM, carbohydrate binding module; CE, carbohydrate esterase; GH, glycoside hydrolases; GT, glycosyltransferase; PL, polysaccharide lyase.

**Figure 2:**
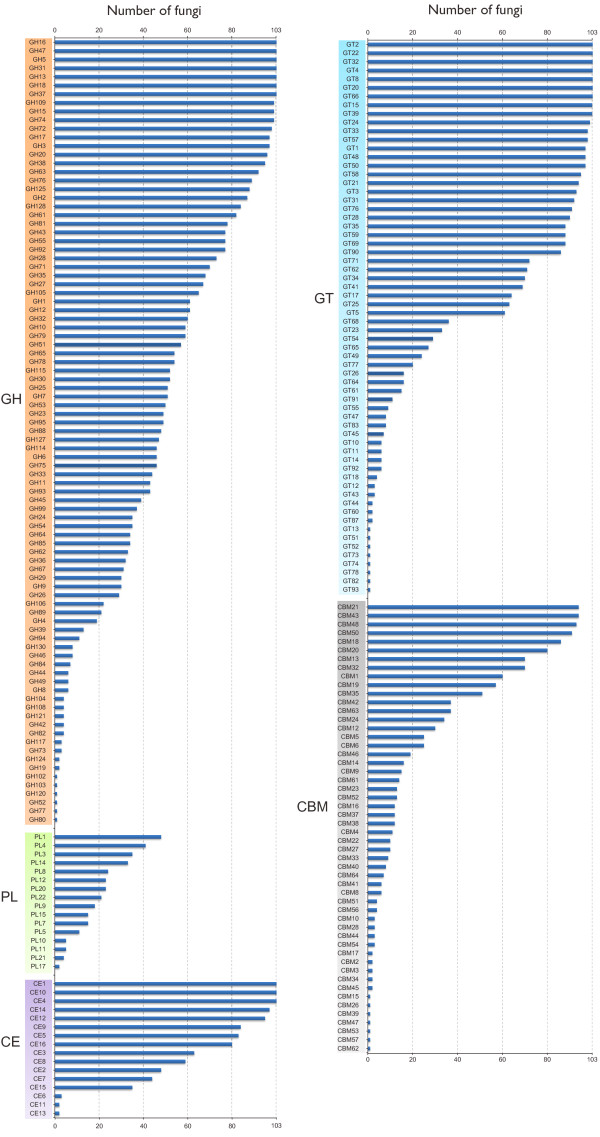
**The distribution of each CAZyme family in 103 fungi. **Blue bars showed the numbers of fungi that have the members of specific CAZyme family.

In total, 187 CAZyme families were identified in fungal predicted proteomes. Over a half of the fungi analyzed contain more than 300 CAZymes (Figure 1; Additional file 1). Note that the ‘CAZymes’ referred here and below indicates functional modules or domains not genes unless otherwise specified. Some CAZyme families, such as CE1, GH5, GH47, and GT2, were detected in all the fungal species examined (Figure 2), while some others, such as CE13, GH104, GH42, and GH77, occurred only in a few fungi (Enzymatic activities are listed in Additional file 2). Interestingly, the distribution of some CAZyme families appeared to be phylum-specific. For example, 28 families, including GH130, GH67, GH94, PL10, and PL11, were only found in the Ascomycetes. In contrast, 15 families, including GH44 and PL15, appeared to be *Basidiomycota*-specific (Table 1).

**Table 1:** Phylum specific CAZyme and CBM families

**Family**	***A***	***B***	***Z***	***C***	**Family**	***A***	***B***	***Z***	***C***
CBM15	0	0	1	0	GH44	0	6	0	0
CBM16	12	0	0	0	GH49	6	0	0	0
CBM23	13	0	0	0	GH52	0	0	1	0
CBM26	1	0	0	0	GH67	31	0	0	0
CBM27	10	0	0	0	GH77	0	0	0	1
CBM28	3	0	0	0	GH80	0	1	0	0
CBM37	12	0	0	0	GH82	4	0	0	0
CBM39	0	1	0	0	GH94	11	0	0	0
CBM44	3	0	0	0	GT13	0	1	0	0
CBM47	0	1	0	0	GT18	0	4	0	0
CBM53	0	0	0	1	GT43	0	3	0	0
CBM57	1	0	0	0	GT51	1	0	0	0
CBM62	1	0	0	0	GT52	0	1	0	0
CBM8	0	6	0	0	GT54	29	0	0	0
CBM9	15	0	0	0	GT55	9	0	0	0
CE11	2	0	0	0	GT73	0	1	0	0
CE6	0	0	3	0	GT74	0	1	0	0
GH102	0	1	0	0	GT78	1	0	0	0
GH103	1	0	0	0	GT82	1	0	0	0
GH117	3	0	0	0	GT93	0	1	0	0
GH120	1	0	0	0	PL10	5	0	0	0
GH121	4	0	0	0	PL11	5	0	0	0
GH124	0	1	0	0	PL15	0	15	0	0
GH130	8	0	0	0	PL17	2	0	0	0

Digits refer to the number of fungi which have enzymes in specific family.

*A*, *Ascomycota*; *B*, *Basidiomycota*; *Z*, *Zygomycota*; *C*, *Chytridiomycota*.

#### Glycoside hydrolases (GHs)

**Figure 3:**
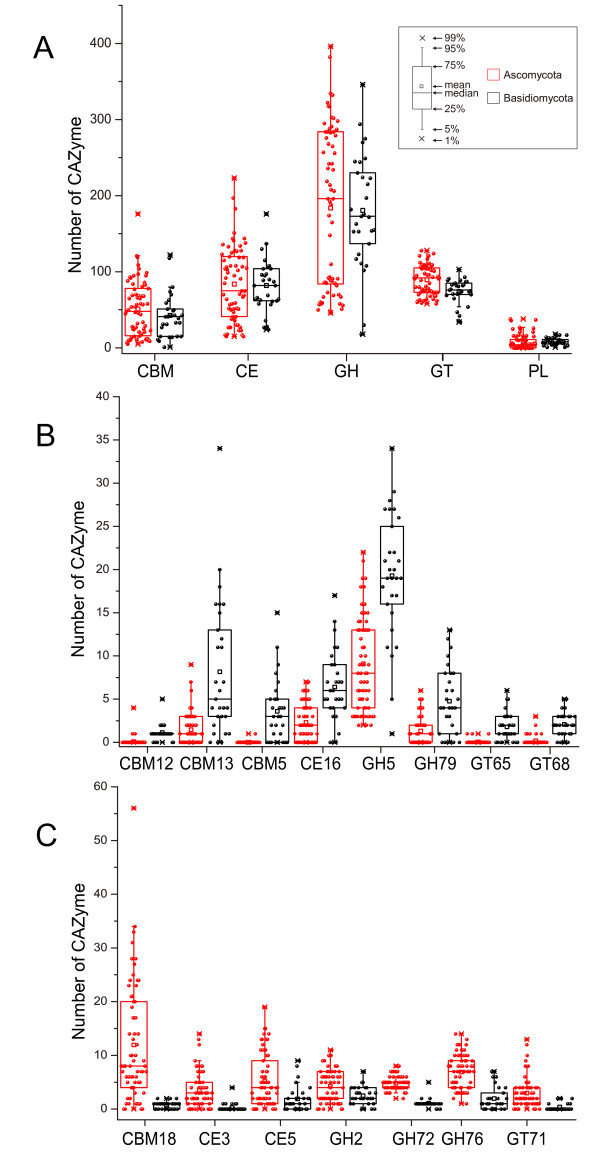
**Different numbers of CAZymes between *Ascomycetes *and *Basidiomycetes*. **(**A**) The number of CAZymes in each class and CBMs were plotted for *Ascomycetes *and *Basidiomycetes*. (**B**) The number of CAZymes in the families which were more abundant in *Basidiomycetes* than in *Ascomycetes *(*t* test, P < 0.01). (**C**) The number of CAZymes in the families which were more abundant in *Ascomycetes *than in *Basidiomycetes *(*t* test, P < 0.01). See Figure 1 for abbreviations.

GHs hydrolyze the glycosidic bond between two or more carbohydrates, or between a carbohydrate and a non-carbohydrate moiety, such as a protein, or a lipid [1]. To date, GHs are grouped into 127 families based on amino acid sequence in the CAZy database. Among the 127 families, 91 of them were detected in fungi examined, with the most prevalent families being GH5, GH13, GH31, and GH61 (Figure 2). Our results showed that GH families vary distinctly on distribution and abundance in fungi (Figure 2). For example, numerous members of families GH16 and GH18 are present in all fungi examined and 102 fungi, respectively. For families GH73, GH77, and GH104, only a single member each was identified in one predicted proteome (Figure 2). Interestingly, only the entomopathogenic fungus *Cordyceps militaris* and symbiotic fungus *Laccaria amethystina* have one member of family GH19, which is expanded in plants and bacteria [1,22]. Ascomycetes and Basidiomycetes differ in the abundance of some families. For instance, Ascomycetes have more members of families GH2 (independent samples *t* test, P < 0.01), GH72 (P < 0.01), and GH76 (P < 0.01) but fewer members of families GH5 (P < 0.01) and GH79 (P < 0.01) (Figure 3) than Basidiomycetes.

#### Polysaccharide lyases (PLs)

PLs mainly degrade glycosaminoglycans and pectin [1,23]. They are classified into 21 families in CAZy database. Our results showed that fungi encode 16 PL families, with the most populated family being PL1 (Figure 2). Ascomycetes and Basidiomycetes have no obvious differences in the number of PLs. However, families PL10, PL11, and PL17 are *Ascomycota*-specific although they are present only in few Ascomycetes. Some families, such as family PL15, are specific to *Basidiomycota* (Table 1). Among the 103 fungi examined, 21 lack any PL. The majority of them are saprophytic or facultative parasitic, such as yeasts and fungi in genus *Trichophyton*. The biotrophic barley powdery mildew fungus *Blumeria graminis* is the only plant pathogenic fungus that lacks any PL.

#### Carbohydrate esterases (CEs)

CEs catalyze the de-O or de-N-acylation of esters or amides and other substituted saccharides in which sugars play the role of alcohol and amine [24]. Our results showed that fungi have 15 of the 16 CE families, with family CE11 being the only one missing. The necrotrophic pea root pathogen *Nectria haematococca* has the most CEs (223). In general, Ascomycetes and Basidiomycetes have similar numbers of CEs, whereas Ascomycetes have more members of families CE3 (P < 0.01) and CE5 (P < 0.01) but fewer members of family CE16 (P < 0.01) than Basidiomycetes (Figure 3). Families CE1 and CE10 are present in all the fungi examined and family CE4 is absent only in the nematophagous facultative parasitic fungus *Arthrobotrys oligospora*. In contrast, families CE6 and CE13 were found only in 3 and 2 fungi, respectively (Figure 2). Members of families CE1 and CE10 share the common activities of carboxylesterase and endo-1,4-β-xylanase. However, they have a great diversity in substrate specificity. For example, vast majority of CE10 enzymes act on non-carbohydrate substrates [1].

#### Carbohydrate-binding modules (CBMs)

CBMs are appended to carbohydrate active enzymes that degrade insoluble polysaccharides [25]. Fifty two of 65 CBM families were detected in fungi examined, with the most prevalent families being CBM21 and CBM23 (Figure 2). Ascomycetes tend to have more CBM18 (P < 0.01) domains but fewer CBM12 (P < 0.01), CBM13 (P < 0.01) and CBM5 (P < 0.01) domains than Basidiomycetes (Figure 3). Furthermore, many CBM families tend to be *Ascomycota*-specific, such as CBM16, CBM23, CBM27, and CBM37 (Table 1). Surprisingly, the facultative parasitic fungus *A*. *oligospora* has the most CBMs, particularly CBM1 modules with the putative cellulose -binding function [1].

### Plant cell wall degrading enzymes

Plant cell walls are comprised mainly of pectins, celluloses, hemicelluloses, ligins, and other polysaccharides and proteins. We focus our detailed analysis on pectinases, cellulases, and hemicellulases because they are the major plant cell wall degrading enzymes in fungal pathogens. Although strictly speaking they are not cell wall degrading enzymes, cutinases are also included in this section because they are often produced in early infection stages by phytopathogenic fungi to breach the plant cuticle and function as important virulence factors in some fungi [26].

#### Pectin degrading enzymes (Pectinases)

Pectin can be broken down by pectin lyase, pectate lyase, pectin esterase, and polygalacturonase (PGA) [20,27]. These enzymes mainly fall into nine CAZyme families, including CE8, PL1, PL2, PL3, PL9, PL10, GH28, GH78, and GH88 [1,4,28]. Our results showed that fungi lack PL2 enzymes. Among 67 Ascomycetes examined, 31% (21/67) lack enzymes belonging to these 9 CAZyme families. In contrast, the only one basidiomycete lacks any of them is *Malassezia globosa*, which is a facultative parasitic fungus causing dandruff on human skin. In lower fungi, only the saprophytic chytrid *Allomyces macrogynus* has pectinases. Interestingly, many vascular wilt and root pathogens, such as *Verticillium albo-atrum, Verticillium dahlia, N*. *haematococca*, and *Fusarium oxysporum*, tend to have more pectinases, which may be related to the blockage or collapse of vascular bundles during disease development.

Polygalacturonases (family GH28) play a critical role in pectin degradation in fungal pathogens. Several fungi, such as the necrotrophic white mold fungus *Sclerotinia sclerotiorum*, gray mold fungus *Botryotinia fuckeliana*, and opportunistic human pathogen *Rhizopus oryzae*, have an expanded family of PGAs (see Additional files 3, 4 and 5), suggesting that these fungi have high capacity of pectin degradation. In contrast, all the Saccharomycetes and Schizosaccharomycetes lack any PGA except the budding yeast *Saccharomyces cerevisiae*, which has a single PGA gene. Pectinesterases (family CE8) catalyze the de-esterification of pectin to pectate and methanol. Most fungi contain only a small number (no more than 8) of pectin esterases, which may play an auxiliary role in pectin breaking down.

#### Lignocellulose degrading enzymes (lignocellulases)

Lignocellulose is a tight complex formed by cellulose, hemicellulose, and lignin, and is the most abundant plant biomass on the planet. Lignocellulose degradation is a complex process involving the cooperation of heterogeneous groups of enzymes. For example, the thorough degradation of cellulose requires the collaboration of endoglucanase, cellobiohydrolase, and β-1, 4-glucosidase [29-31]. The GH class contributes the most catalytic enzymes to the degradation of lignocelluloses [3], such as cellulases in families GH1, GH3, GH5, GH45, and GH74 [1,4,31], xylanases in families GH3, GH10, GH11, and GH39 [1,32]. At least 29 GH families are known to be involved in the degradation of plant biomass [1,4,28,31] (Table 2). Among them, families GH2, GH3, GH5, GH27, GH31, GH35, GH43, GH74, and GH78 tend to be more populated or abundant since that they are present in over a half of fungi examined and some of them are expanded in many fungi (Figure 2; Additional files 3, 4 and 5). Our results showed that all fungi examined have cellulose degrading enzymes such as members of family GH74. In contrast, only 38% of the bacterial genomes were reported to code cellulase genes [33].

**Table 2:** Known substrates (most common forms) of CAZymes

**Family**	**Substrates**	**Family**	**Substrates**
GH1	CW (β-glycans)	GH49	ESR (dextran)
GH10	PCW (hemicellulose)	GH5	CW (β-glycans)
GH105	PCW (pectin)	GH51	PCW (hemicellulose)
GH11	PCW (hemicellulose)	GH53	PCW (hemicellulose)
GH115	PCW (hemicellulose)	GH54	PCW (hemicellulose)
GH12	PCW (cellulose)	GH55	FCW (β-1,3-glucan)
GH125	PG (N-glycans)	GH6	PCW (cellulose)
GH13	FCW + ESR (α-glucans)	GH61*	PCW (cellulose)
GH15	ESR (α-glucans)	GH62	PCW (hemicellulose)
GH16	FCW (β-glycans)	GH63	PG (N-glycans)
GH17	FCW (β-1,3-glucan)	GH64	CW (β-1,3-glucan)
GH18	FCW (chitin)	GH65	ESR (trehalose)
GH2	CW (β-glycans)	GH67	PCW (hemicellulose)
GH20	FCW (chitin)	GH7	PCW (cellulose)
GH23	BPG	GH71	FCW (β-1,3-glucan)
GH24	BPG	GH72	FCW (β-1,3-glucan)
GH25	BPG	GH74	PCW (cellulose)
GH26	BPG	GH75	FCW (chitin)
GH27	PCW (hemicellulose)	GH76	FCW (chitin)
GH28	PCW (pectin)	GH78	PCW (pectin)
GH29	PCW (hemicellulose)	GH8	CW
GH3	CW (β-glycans)	GH81	FCW (β-1,3-glucan)
GH30	FCW	GH85	FCW
GH31	PG + ESR + PCW (hemicellulose)	GH88	PCW (pectin)
GH32	ESR (sucrose/inulin)	GH89	FCW
GH35	PCW (hemicellulose)	GH9	CW
GH36	PCW (hemicellulose)	GH93	PCW (hemicellulose)
GH37	ESR (trehalose)	GH94	PCW (cellulose)
GH38	PG (N-/O-glycans)	PL1	PCW (pectin)
GH39	PCW (hemicellulose)	PL11	PCW (pectin)
GH4	ESR	PL14	BEPS
GH43	PCW (pectin + hemicellulose)	PL3	PCW (pectin)
GH45	PCW (cellulose)	PL4	PCW (pectin)
GH46	FCW (chitin)	PL7	BEPS
GH47	PG (N-/O-glycans)	PL9	PCW (pectin)

BEPS: Bacterial exopolysaccharides; BPG: Bacterial peptidoglycan; CW: Cell wall; ESR, energy storage and recovery; PCW: Plant cell wall; PG, protein glycosylation; FCW: Fungal cell wall.

*: Enzymes of GH61 family were not truly glycosidase, however, they are important to degrade lignocellulose in conjunction with cellulases [34], and were kept as GH in the CAZy classification [1].

GH3 family: Enzymes of this family are classified based on substrate specificity into β-D-glucosidases, α-L-arabinofuranosidases, β-D-xylopyranosidases, and N-acetyl-β-D-glucosaminidases [35]. The most common form is β-D-glucosidase [4,28]. Our results showed that GH3 enzymes were abundant in 97 of all fungi examined (Figure 2). Two necrotrophic fungi, *N*. *haematococca* and *F*. *oxysporum*, have more GH3 enzymes (38 and 32, respectively) than any other fungi. The tomato leaf mold fungus *Cladosporium fulvum* is the only biotrophic fungus with a larger number (20) of GH3 enzymes. Among the Chytridiomycetes, only the amphibian pathogen *Spizellomyces punctatus* has the GH3 member.

GH5 family: This is one of the largest GH families. It consists of a wide range of enzymes acting on different substrates [36], with the most common forms being exo-/endo-glucanases and endomannanases [37,38]. Among all the GHs, members belonging to the GH5 family are the most common ones and they are present in all fungi examined, suggesting that these enzymes play important roles in fungal degradation of lignocellulose. In general, Basidiomycetes tend to have more GH5 enzymes than Ascomycetes. The saprophytic basidiomycete *Jaapia argillacea* has the largest number (34) of GH5 enzymes, closely followed by another saprophytic fungus *Botryobasidium botryosum* (29). Interestingly, the biotrophic wheat rust fungi *Puccinia graminis* and *Puccinia triticina* tend to have more GH5 enzymes (28 and 27, respectively) than other fungi. Another biotrophic rust fungus *Melampsora laricis-populina* also has a large number (21) of GH5 enzymes. *Rhodotorula glutinis*, a saprophytic basidiomycete, is the only fungus with a single GH5 member.

GH10 and GH11 families: Enzymes of families GH10 and GH11 both display endoxylanase activities [1,39] but GH10 enzymes have higher substrate specificity than those of family GH11 [38]. Our results showed that 59 and 43 fungi have GH10 and GH11 enzymes, respectively, 44 of 103 fungi examined lack members belonging to these two families, including all entomopathogenic fungi.

GH51 and GH54 families: Enzymes of families GH51 and GH54 mainly decompose hemicelluloses such as arabinoxylan, arabinogalactan, and L-arabinan [1,4,40]. It was reported that fungal α-arabinofuranosidases are mainly found in GH families 51 and 54 [38]. Our results showed that over half of the fungi examined lack either GH51 or GH54 enzymes, and 43 fungi lack any member of these two families (Additional files 3, 4 and 5). The saprophytic fungus *Gymnopus luxurians* has 5 GH51 enzymes, which is more than any other fungi. On the other hand, the facultative parasitic fungus *Penicillium marneffei* tends to have the most GH54 (4) enzymes.

GH55, GH64, and GH81 families: Enzymes of families GH55, GH64, and GH81 display β-1,3-glucanase activities [1,41]. Their known substrates are mainly the fungal cell wall, which is enriched by β-1,3-glucan [11,41]. Callose is also a polysaccharide of β-1,3-glucan in plant cell wall. It is involved in the plant defense responses during interaction with pathogenic fungi [10]. Hence, we also investigated the variety of enzymes belonging to these three families among plant pathogenic fungi. Our results showed that family GH55 and GH81 enzymes showed no obvious variety among plant pathogenic fungi. Five out of six biotrophic fungi lack any GH64 member, whereas only two necrotrophic and one hemibiotrophic fungi lack any. Interestingly, the hemibiotrophic fungus *Moniliophthora perniciosa* lacks any member of these three families, distinctly deviating from other hemibiotrophic fungi.

#### Cutinases

Cutin is composed of hydroxy and hydroxyepoxy fatty acids. Cutinases (family CE5) catalyze the cleavage of ester bonds of cutin to release cutin monomers. Among the 103 fungi analyzed, 83 of them have cutinases (Figure 2). In seven lower fungi belonging to *Zygomycota* and *Chytridiomycota*, only the zygomycete *R*. *oryzae* has no cutinase. Interestingly, the necrotrophic fungus *Fomitiporia mediterranea* lacks any cutinase. The hemibiotrophic rice blast fungus *Magnaporthe oryzae* has 19 cutinases, which is more than any other fungus. In *M. oryzae*, at least one cutinase gene is known to be important for plant infection [42,43]. Both two necrotrophic fungi, *Gaeumannomyces graminis* and *V*. *albo-atrum,* have 15 cutinases. Interestingly, all biotrophic fungi have cutinases, in contrast to that two symbiotic fungi, *Paxillus involutus* and *Paxillus rubicundulus*, lack any.

### Comparing abundance of CAZymes among fungi

The fungi examined in this study vary significantly in the number of CAZymes. For example, nineteen of them have more than 500 CAZymes, twenty-two fungi have fewer than 200 CAZymes. In general, Dothideomycetes and Sordariomycetes contain more CAZymes and Saccharomycetes and Schizosaccharomycetes have fewer (Figure 1). For instance, there are 730 and 125 CAZymes in *F. oxysporum* and *Schizosaccharomyces cryophilus*, respectively.

#### Saprophytic fungi

Based on the number of predicted CAZymes, saprophytic fungi can be divided into two groups. The first group consists of 24 fungi that have more than 200 CAZymes from classes GH, CE and PL. However, they lost several CAZyme families, including families CE11, GH73, GH80, and GH82. Fungi of the second group, including 4 Schizosaccharomycetes, 2 Saccharomycetes, one eurotiomycete *Uncinocarpus reesii,* and one basidiomycete *R*. *glutinis*, have fewer than 200 CAZymes, which is fewer than the other saprophytes (Figure 1). Only *R*. *glutinis* of this group has PLs (Additional file 3). In contrast to the first group, the latter lost many CAZyme families of GHs and CEs, including families CE7, CE8, GH1, GH6, GH10, GH11, GH30, and GH79.

#### Facultative parasitic fungi

Facultative parasitic fungi normally live as saprobes but they are opportunistic pathogens of plants or animals. Similar to saprophytic fungi, facultative fungal pathogens can be divided into two groups based on the number and type of CAZymes (Figure 1). The ten fungi in the first group have more CAZymes than the second group and mainly are saprobes. Twenty members of the second group have fewer than 230 CAZymes. Most of them lack PL enzymes and are facultative vertebrate pathogenic fungi, such as *Candida* species. In contrast to the first group, they lost most families of CAZymes related to the plant biomass degradation, such as families CE5, GH6, GH7, GH10, GH12, GH36, GH53, GH54, GH62, PL1, and PL3 (Additional file 4).

#### Obligate parasitic fungi

Obligate parasitic fungi depend on the presence of plant or animal hosts to complete their life cycle. In comparison with hemibiotrophic fungi, biotrophic fungi have the least CAZymes and necrotrophic fungi have the most CAZymes (Figure 1), although the numbers and variety of CAZymes in each group are diverse.

**Figure 4:**
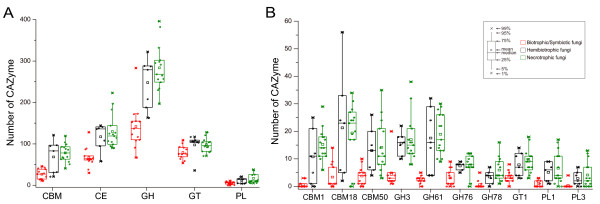
**Comparison of CAZymes in different plant pathogenic fungi. **(**A**) The number of CAZymes in each class and CBMs were plotted for biotrophic (red), hemibiotrophic (black), and necrotrophic (green) pathogens. (**B**) The number of CAZymes in the families that showed significant differences among biotrophic, hemibiotrophic, and necrotrophic pathogens. Differentiation was support by the *t *test at the significant level with P < 0.01. See Figure 1 for abbreviations.

Biotrophic fungi derive nutrients from living tissues. Four of six biotrophic fungi analyzed are in phylum *Basidiomycota*, two are in phylum *Ascomycota*. In contrast to necrotrophic and hemibiotrophic fungi, biotrophic fungi lack GH6 enzymes, which are known to display endoglucanase and cellobiohydrolase activities [1] for plant cell wall degradation [4,28]. In general, biotrophic fungi tend to have fewer plant cell wall degrading enzymes than necrotrophic and hemibiotrophic fungi, such as enzymes of GH61, GH78, PL1 and PL3 (Figure 4). Furthermore, they also have fewer enzymes belonging to family GH76 and CBM1, CBM18, and CBM50 (Figure 4). Interestingly, CBM18 domains are present in various enzymes from families GH18, GH19, GH23, GH24, GH25, and GH73 [1]. Although it lacks experimental supports, the absence or reduction of these families may be correlated to their biotrophic lifestyles. Unlike other members of this group, the barley powdery mildew fungus *B*. *graminis* lacks any PL enzyme. *C. fulvum* differs from most other members of *Mycosphaerellaceae* by being a biotroph, while the others are hemibiotrophs or necrotrophs [44]. Interestingly, our results showed *C*. *fulvum* has significantly more CWDEs of families GH3, GH31, GH43, and PL3 than any other biotrophic fungi (Additional file 5). Similar to biotrophic pathogens, symbiotic fungi contain small number of CAZymes and also lack enzymes of family GH6. For example, *Laccaria bicolor*, a member of the *Tricholomataceae* family that can develop symbiotic associations with plant roots [45], contains a small number of CAZymes (Additional file 5). It may be beneficial to symbiotic fungi to contain fewer CAZymes for its symbiotic association with host plants.

Necrotrophic plant pathogens derive nutrients from dead host cells. Most of the necrotrophic fungi sequenced to date are from phyla *Sordariomycetes*, *Dothideomycetes*, and *Leotiomycetes*. The wood rotting fungus *Dichomitus squalens* is the only member of this group that lacks any PL1 enzymes which expanded in other necrotrophic fungi. *G*. *graminis*, *M*. *poae*, and *S*. *sclerotiorum* have fewer PLs than other fungi in this group (Figure 1; Additional file 5). Hemibiotrophic fungi have the initial biotrophic phase but switched to necrotrophic growth at late infection stages. In general, these fungi have more CAZymes than biotrophic fungi but similar to necrotrophic fungi in the number and diversity of CAZymes. *M*. *perniciosa,* the causal agent of the witches’ broom disease of cocoa, contains only two cutinases (CE5), which is fewer than any other hemibiotrophic fungi. The diversity of CAZymes in fungi with different lifestyles suggests that the adaptation of fungal pathogens to different plant biomass and degrading capabilities.

### The diversity of CAZymes between monocot and dicot pathogens

**Figure 5:**
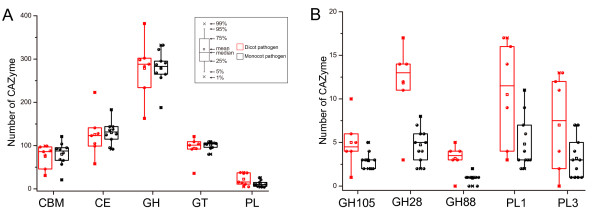
**Comparison of CAZymes among fungi pathogenic to monocots and dicots. **(**A**) The number of members in each CAZyme class and CBMs in the predicted proteomes were plotted for fungi that infect dicots (red dots) or monocots (black dots). (**B**) The number of CAZymes which showed obvious differentiation among fungi that infect dicots or monocots. Dicot pathogens have more pectinases from families GH28 (P < 0.01), GH88 (P < 0.01) and GH105 (P < 0.01) than fungi pathogenic to monocots. Although the significance of the comparison was not supported by the *t *test (P > 0.05), some dicot pathogens tend to have much more PL1 and PL3 enzymes than monocot pathogens. See Figure 1 for abbreviations.

Some fungi can infect both dicots and monocots such as *P. graminis*, *Melampsora larici-populin*, and *F*. *oxysporum*. However, many fungi can only infect either dicots or monocots, such as *P. teres*[46]. Cell wall components of dicots and monocots are different, especially in the proportion of pectin and hemicellulose [5,20]. Activities of plant biomass degrading enzymes in some fungi also are known to have preference of biomass type of monocot or dicot plants [5]. To detect whether the CAZyme family diversity is correlated to the specificity of their hosts, we compared different pathogens that infect monocots or dicots. Because biotrophic fungi lack most of plant cell wall degrading enzymes, they were excluded in this analysis. In general, dicot pathogens have more pectinases belonging to families GH28 (P < 0.01), GH88 (P < 0.01), and GH105 (P < 0.01) than fungi pathogenic to monocots (Figure 5), which agrees with the fact that cell walls of dicots are composed of higher levels of pectin than monocots [19]. Although the significance of the comparison between monocot and dicot pathogens with family PL1 and PL3 were not supported by the *t* test, some dicot pathogens, such as *N*. *haematococca*, *V*. *albo*-*atrum*, and *V*. *dahlia*, have more PL1 and PL3 enzymes than monocot pathogens.

Although dicot and monocot plants have different amounts of hemicelluloses in their cell wall, their pathogens have no significant differences in the diversity or number of enzymes related to the hemicellulose degrading. It should be noted that dicots and monocots have different levels of xylans and mannans in the primary cell wall but approximately the same level in the secondary cell wall [19]. The number of enzymes involved in cellulose degradation also has no significant differences between dicot and monocot pathogens, which agree with the fact that dicots and monocots are composed of similar levels of cutin and cellulose.

### Saprophytic fungi have fewer CAZymes than plant pathogenic fungi

**Figure 6:**
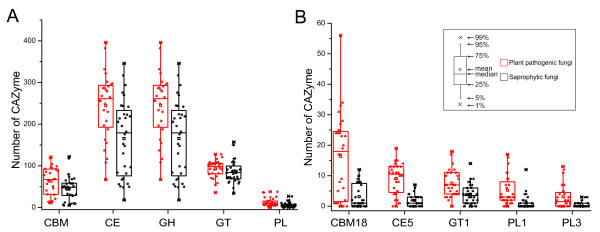
**Comparison of CAZymes between saprophytic fungi and plant pathogenic fungi. **(**A**) The number of members in each CAZyme class and CBM in the predicted proteomes were plotted for plant pathogenic fungi (red dots) and saprophytic fungi (black dots). (**B**) The number of CAZymes which showed obvious differentiation supported by *t *test among plant pathogenic fungi and saprophytic fungi.

In general, saprophytic fungi are considered to produce a variety of CAZymes. Our results showed that saprophytic fungi have fewer CAZymes belonging to classes CE (P < 0.05), GH (P < 0.05), and PL (P < 0.05), particularly families CE5 (P < 0.01), GT1 (P < 0.01), PL1 (P < 0.01), and PL3 (P < 0.01), than plant pathogenic fungi (Figure 6).

Interestingly, some fungi known for high capability of plant biomass degradation were found to contain fewer plant cell wall degrading enzymes in our analysis. For example, the opportunistic human pathogen *R. oryzae* has fewer lignocelluosic biomass degrading enzymes although it is known for its high degrading capacity [11]. The high level of gene expression and enzyme activity may contribute to these fungi’s ability to degrade plant biomass.

### The presence of plant cell wall degrading enzymes in fungi associated with animals

Some bacteria do not have a saprophytic lifestyle or cellulose degrading activity but contain cellulases belong to family GH6, GH12, and GH5 [33]. One example is the animal and human tuberculosis pathogen *Mycobacterium tuberculosis*, which has two cellulase genes belonging to family GH6 and GH12 but has no known relationship with plants [33]. Our results showed that some fungal parasites of vertebrates in group 2 of facultative parasitic fungi have lignocellulases although they can live only as saprobes or parasites to animals. For example, *M*. *globosa* is a lipid-dependent microorganism responsible for the onset of dandruff and other skin conditions in humans [47]. It has one member of family GH74, which is known to be involved in the degradation of plant celluloses [4,28]. Furthermore, it contains members of families GH105, GH31, GH43, GH5, and GH8, with which substrates are mainly plant lignocelluloses [4,28]. The amphibian pathogen *Batrachochytrium dendrobatidis* grows on amphibian skin [48] also has lignocellulases and is not known for association with plants.

Unlike bacteria, these fungi produce enzymes belong to families GH5 and GH3 but not families GH6 and GH12. Whereas GH5 enzymes mainly include endo/exo-glucanase, endo-1,6-glucanase, mannanase, and xylanase, GH3 enzymes include β-glucosidase, glucan β-1,3-glucosidase, cellodextrinase, and exo-1,3-1,4-glucanase. In contrast, GH6 enzymes include endoglucanase and cellobiohydrolyase and GH12 enzymes include endoglucanase, xyloglucan hydrolase, and β-1,3-1,4-glucanase. The predicted GH3 enzyme of *M*. *globosa* has the entire functional domain and conserved aspartic acid residue of the GFVISDW motif [35], suggesting that it is likely active. However, functional domains of some GH5 enzymes in *M*. *globosa*, such as 164657103, 164657414, and 164655644, are truncated and unlikely to be active. Some cellulases are involved in cellulose biosynthesis in bacteria and plants [49,50] but the fungal cell wall lacks cellulose. Thus, cellulase genes in animal pathogenic fungi may be remnants of ancestry genes, indicating that they may be evolved from plant pathogenic fungi.

### The expression profiles of CAZyme genes in *Fusarium graminearum* during plant infection

To investigate whether genes coding CAZymes play important roles during plant infection, we analyzed the microarray data of the hemibiotrophic fungus *F. graminearum* from spike infection of barley (FG1) and wheat head (FG15), as well as conidia germination (FG7) downloaded from PLEXdb database (http://www.plexdb.org). All of the CAZyme genes (CEs, PLs, and GHs) identified in *F. graminearum* were expressed in these experiments. The expression profiles of these genes could be categorized into nine models by k-means clustering algorithm implemented in program Mayday [51]. The expression profiles of spike infection of barley and wheat head were similar to each other but different from those of conidium germination. Most CAZyme genes were up-regulated during plant infection (Additional file 6) but the majority of cluster 9 genes were down-regulated. These genes generally encode CEs and fungal cell wall decomposing enzymes, such as CEs FGSG_03012, FGSG_00784, and FGSG_11578 and GHs FGSG_03827, FGSG_04648, and FGSG_09648 (Additional file 7). In contrast to plant infection, conidium germination showed different gene expression models in cluster 1, 2, 5, and 7. Genes in cluster 1 and 7 were down-regulated during conidium germination, indicating that they play less important roles in this process. Interestingly, genes in cluster 5 were up-regulated during germination but showed no obvious changes during wheat or barley infection, suggesting that they play more important roles in conidium germination than in plant infection.

### Evolution of fungal polysaccharide lyase family 1 (PL1)

**Figure 7:**
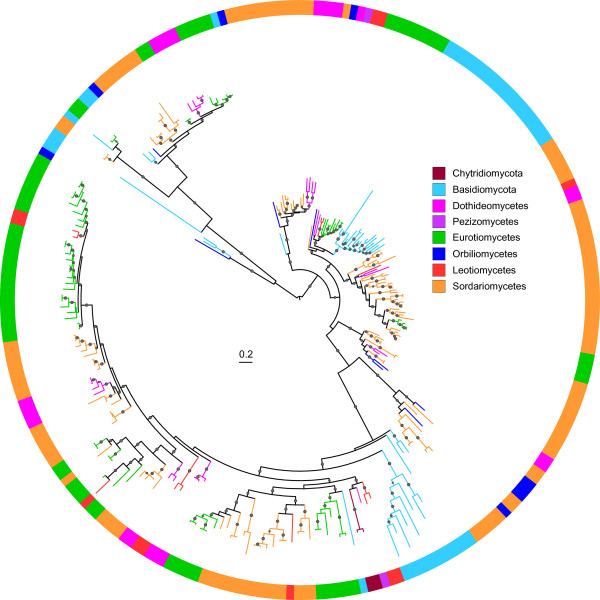
**The maximum likelihood tree of family PL1. **The phylogenetic tree was constructed with PhyML3.0 based on a multiple sequence alignment generated with PSI-Coffee [54]. The midpoint rooted base tree was drawn using Interactive Tree Of Life Version 2.1.1 (http://itol.embl.de/). The p-values of approximate likelihood ratios (SH-aLRT) plotted as circle marks on the branches (only p-values >0.5 are indicated) and circle size is proportional to the p-values. Scale bars correspond to 0.2 amino acid substitutions per site. A detailed version of this tree is found in Additional file 8.

Family PL1 mainly displays activities of pectate lyases and pectin lyases, and is one of the largest families of PL class in fungi. Members in PL1 are important for plant infection and may be related to fungal virulence [17]. We found that saprophytic and pathogenic fungi differ significantly in the number of PL1 enzymes (Figure 6). To investigate their evolution in fungi, we reconstructed the phylogenetic tree for the PL1 enzymes (Figure 7 and Additional file 8). Many clades containing entries from different fungal taxa in the phylogenetic tree, suggesting that the last common fungal ancestor possessed numerous paralogous PL1 genes. The clades contain only one or some of fungal taxa and none of the taxa retains representatives of all ancestral paralogs, indicating that different subsets of ancestral paralogs may have been lost in certain fungal taxa during evolution. For example, Basidiomycetes may have lost most of the ancestral PL1 genes whereas Sordariomycetes have retained most of them (Figure 7). Furthermore, lineage or species-specific gene duplication (gain) events also have occurred within many fungal taxa, particularly in plant pathogens. For example, the *Fusarium* species, which are necrotrophic pathogens, contain many closely related paralogous PL1 genes, suggesting of the recent gene duplication and divergence events. In all, the phyletic distribution and phylogenetic relationship of PL1 genes within different fungal taxa revealed a complex history of lineage-specific gene expansions and attritions which may be related to their nutritional strategies.

## Conclusions

In conclusion, we systemically identified glycoside hydrolases, polysaccharide lyases, carbohydrate esterases, and glycosyltransferases, as well as carbohydrate-binding modules from predicted proteomes of 103 representative fungi belonging to *Ascomycota, Basidiomycota, Chytridiomycota*, and *Zygomycota*. Comparative analysis revealed that fungi exhibit tremendous diversity in the number and variety of CAZymes. Among them, some families of GH and CE are the most prevalent CAZymes that are present in all the fungi analyzed. Importantly, cellulases of some GH families are present in fungi that are not known to degrade celluloses. Our results also showed that plant pathogenic fungi, in general, contain more CAZymes than saprophytic, symbiotic, and animal pathogens. Among plant pathogens, biotrophic fungi have fewer CAZymes in comparison with necrotrophic and hemibiotrophic fungi. In addition, fungi infecting dicots contain more pectinases than fungi infecting monocots. Interestingly, several non-yeast saprophytic fungi, including *R. oryzae,* contain fewer CAZymes although they have high capacity of plant biomass degradation. Furthermore, analysis of the gene expression profiles of the wheat scab fungus *F*. *graminearum* revealed that most of the CAZyme genes were up-regulated during plant infection. Phylogenetic analysis of the PL1 family revealed a complex history of lineage-specific gene expansions and attritions. Results from this study provide insights into the variety and expansion of fungal CAZyme families and revealed the relationships of CAZyme size and diversity of fungi with their nutritional strategy and host specificity.

## Methods

### Data collection and CAZyme annotation

The predicted proteomes of 49 fungi were downloaded from Fungal Genome Initiative (FGI) site of Broad Institute (http://www.broadinstitute.org/science/projects/projects), 30 were obtained from GenBank of NCBI, 23 were downloaded from DOE Joint Genome Institute (JGI) site [52], and 1 was downloaded from BluGen (http://www.blugen.org/) (Additional file 1)*.*

We used the Hmmscan program in HMMER 3.0 package [53] to search each of fungal predicted proteomes with the family-specific HMM profiles of CAZymes downloaded from dbCAN database [21] as queries. The primary results were processed by the hmmscan-parser script supplied by the dbCAN.

### Cluster analysis of gene expression profiles of *Fusarium graminearum* CAZymes

The expression data were downloaded from Plant Expression Database (PLEXdb) (http://www.plexdb.org/index.php). For each experiment, three biological replicates were analyzed. The expression data of RMA treatment means for all probesets were used. The software Mayday 2.13 [51] was used to construct the k-means clustering with Pearson correlation distance measure. The CAZymes in GT class were not included in this analysis.

### Phylogenetic analysis

Multiple alignments of protein sequences were constructed using PSI-Coffee [54] and the regions of large gap and ambiguous alignments were removed manually. Maximum likelihood (ML) phylogeny were estimated with PhyML3.0 assuming 8 categories of γ-distributed substitution rate and SPRs algorithms, based on amino acid sequence alignment and the best-fit model LG + F selected by ProtTest2.4 [55]. The reliability of internal branches was evaluated based on SH-like approximate likelihood ratios (aLRT) supports. The resulting alignment and phylogenetic tree have been deposited in treeBASE under URL (http://purl.org/phylo/treebase/phylows/study/TB2:S13822?x-access-code=5adfa7c8af1e503811a8adfcec7f769f&format=html).

### Statistical analysis

We used the program OriginPro 8.5 (http://originlab.com/index.aspx?go=PRODUCTS/OriginPro) to generate statistical box charts. Independent samples *t* tests were performed by the program SPSS Statistic 17.0 (http://www.01.ibm.com/software/analytics/spss/).

## Abbreviations

CA: Cutinase; CAZyme: Carbohydrate activity enzyme; CBM: Carbohydrate binding module; CE: Carbohydrate esterase; GH: Glycoside hydrolases; GT: Glycosyltransferase; CWDE: Cell wall degrading enzyme; PGA: Polygalacturonase; PL: Polysaccharide lyase.

## Competing interests

The authors declare that they have no competing interests.

## Authors’ contributions

ZZ performed bioinformatic analyses, participated in the interpretation of the results and drafted the manuscript. HL designed and coordinated the study, participated in the bioinformatic analyses, in the interpretation of the results and in the writing of the manuscript. CW participated in the coordination of the study. JRX conceived the study, participated in the interpretation of the results and in the writing of the manuscript. All authors read, corrected and approved the final manuscript.

## Supplementary Material

Additional file 1 Distribution of CAZymes in 103 fungi. Click here for file

Additional file 2 Known activities of each enzyme/domain family. Click here for file

Additional file 3 Distribution of CAZymes and CBMs in saprophytic fungi. Click here for file

Additional file 4 Distribution of CAZymes and CBMs in facultative pathogenic fungi. Click here for file

Additional file 5 Distribution of CAZymes and CBMs in plant pathogenic fungi. Click here for file

Additional file 6 **Cluster profiles of *Fusarium graminearum *CAZyme genes during infection of wheat or barley and conidium germination.** Click here for file

Additional file 7 Expression changes of Fusarium graminearum CAZyme genes during wheat infection, barley infection, and conidium germination. Click here for file

Additional file 8 The maximum likelihood tree of family PL1. Click here for file
