# Supplementary material for: Comparative analysis of fungal genomes reveals different plant cell wall degrading capacity in fungi
Source: BMC Genomics. 2013 Apr 23;14:274. doi: 10.1186/1471-2164-14-274 (PMC3652786; doi:10.1186/1471-2164-14-274)
Supplement: Additional file 6 — Cluster profiles of Fusarium graminearum CAZyme genes during infection of wheat or barley and conidium germination. [file 1471-2164-14-274-S6.pdf]

Additional file 6. Cluster profiles of *Fusarium graminearum* CAZyme genes during infection of wheat or barley and conidium germination. All time-series have been transformed to (0, v_1−v_0,..., v_n−v_0) so that the time series starts at 0. The software Mayday 2.13 [50] was used to construct the k-means clustering with Pearson correlation distance measure and 9 clusters. FG1 and FG15 were expression profiles of *F. graminearum* CAZyme genes during infection of barley spikes and wheat heads, respectively. FG7 panels were expression profiles of these genes during conidium germination.

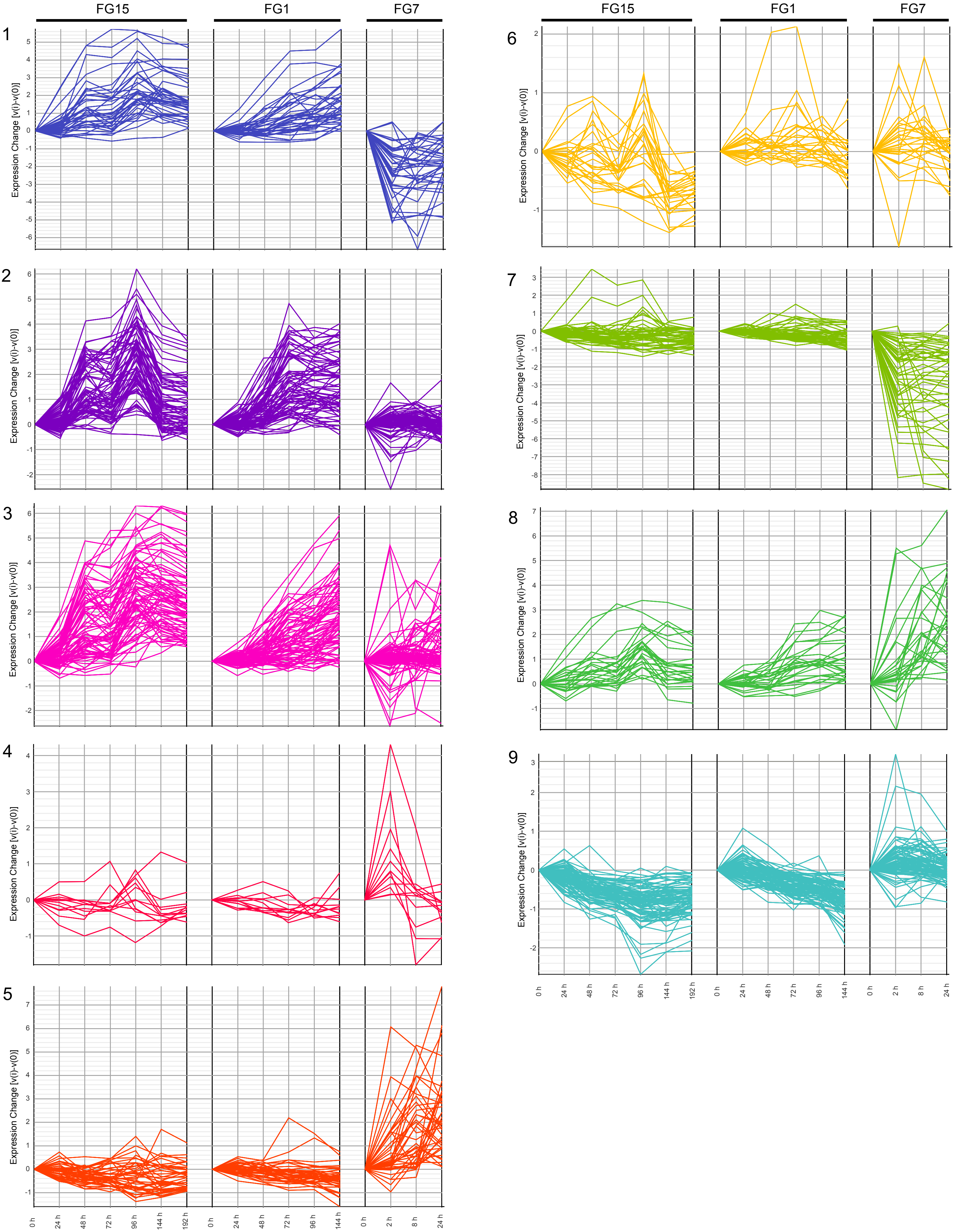
FG15
FG1
FG7
1
2
3
4
5
6
7
8
9
Expression Change [v(i)-v(0)]
0 h
24 h
48 h
72 h
96 h
144 h
192 h
2 h
8 h
