# Supplementary material for: Comparative analysis of fungal genomes reveals different plant cell wall degrading capacity in fungi
Source: BMC Genomics. 2013 Apr 23;14:274. doi: 10.1186/1471-2164-14-274 (PMC3652786; doi:10.1186/1471-2164-14-274)
Supplement: Additional file 8 — The maximum likelihood tree of family PL1. [file 1471-2164-14-274-S8.pdf]

Additional file 8. The maximum likelihood tree of family PL1. The phylogenetic tree was constructed with PhyML3.0 based on a multiple sequence alignment generated with PSI-Coffee [53]. The midpoint rooted base tree was drawn using Interactive Tree Of Life Version 2.1.1 (http://itol.embl.de/). The p-values of approximate likelihood ratios (SH-aLRT) plotted as circle marks on the branches (only p-values >0.5 are indicated) and circle size is proportional to the p-values. Scale bars correspond to 0.2 amino acid substitutions per site.

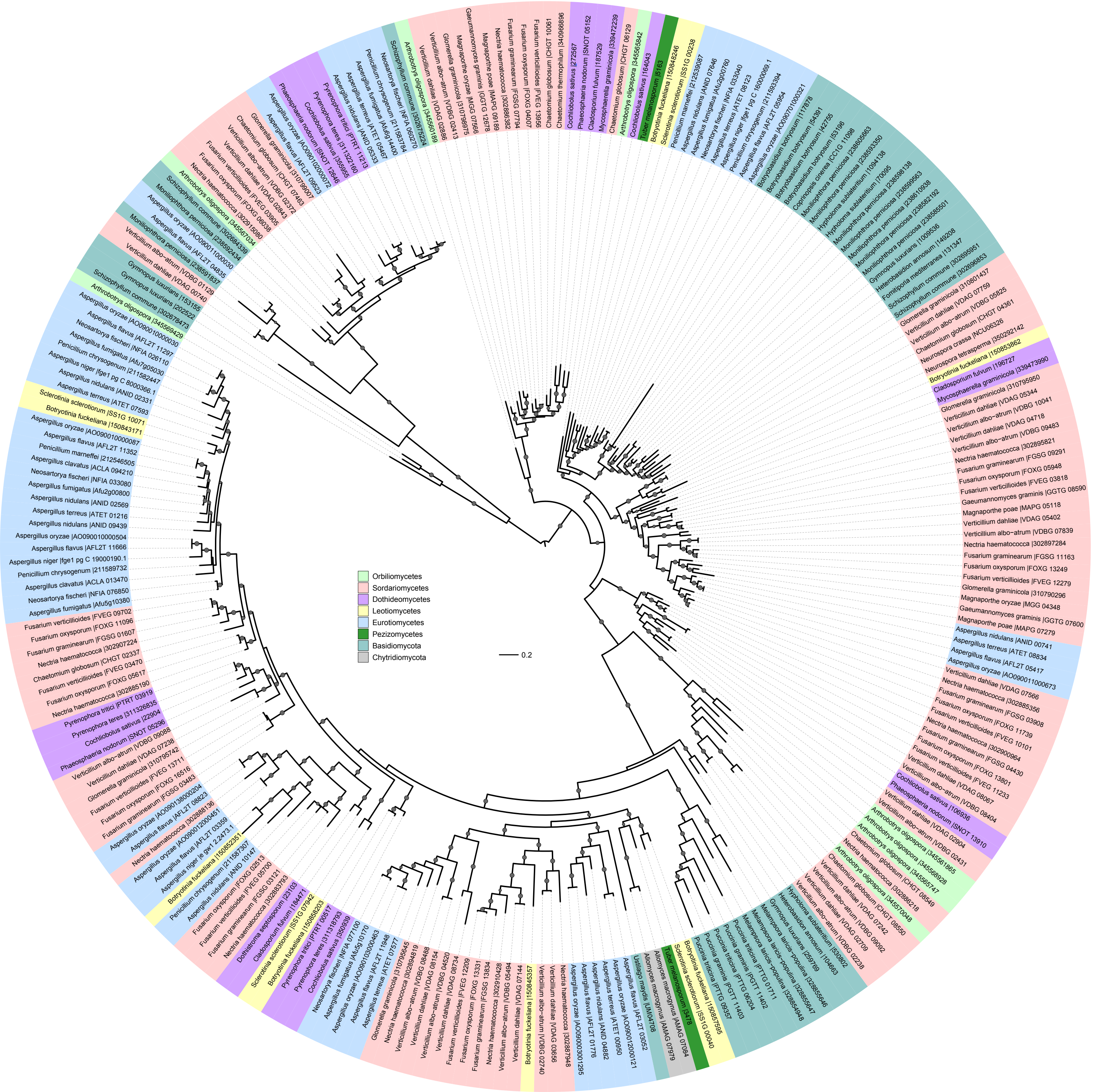
Orbiliomycetes
Sordariomycetes
Dothideomycetes
Leotiomycetes
Eurotiomycetes
Pezizomycetes
Basidiomycota
Chytridiomycota
0.2
